# Supplementary material for: Prognostic Factors for Survival in Glioblastoma: A Retrospective Analysis Focused on the Role of Hemoglobin
Source: Biomedicines. 2024 May 29;12(6):1210. doi: 10.3390/biomedicines12061210 (PMC11200864; doi:10.3390/biomedicines12061210)
Supplement: Supplementary file 1 [file biomedicines-12-01210-s001.zip › biomedicines-2992917-supplementary.pdf]

Prognostic Factors for Survival in Glioblastoma: A Retrospective Analysis Focused on the Role of Hemoglobin

SUPLLEMENTARY MATERIAL

Table S1. Results of the Cox multivariate analyses focused on the genetic and other factors with continuous values.

Factor	β	SE	Wald	*p*	HR	$^{0.95}CI_{LB}$	$^{0.95}CI_{UB}$
Age	0.046	0.011	16.452	<0.001	1.047	1.024	1.071
HIF-1α	0.123	0.053	5.349	0.021	1.131	1.019	1.256
HIF-1β	-0.012	0.005	5.109	0.024	0.988	0.978	0.998
GFAP	-0.015	0.009	2.565	0.109	0.985	0.968	1.003
CD34	0.013	0.009	1.880	0.170	1.013	0.995	1.031
CD44	0.009	0.007	1.881	0.170	1.009	0.996	1.023
D240	0.005	0.004	1.435	0.231	1.005	0.997	1.013
Vim	0.006	0.006	0.865	0.352	1.006	0.994	1.018
SOX11	0.006	0.006	0.835	0.361	1.006	0.994	1.018
MerTK	-0.005	0.006	0.677	0.411	0.995	0.983	1.007
STAT3	-0.002	0.004	0.226	0.635	0.998	0.990	1.006
Hb	-0.003	0.009	0.141	0.707	0.997	0.980	1.014
Olig2	-0.001	0.005	0.067	0.796	0.999	0.990	1.008
PDGFR	0.000	0.004	0.005	0.942	1.000	0.991	1.008
Meox2	0.000	0.005	0.001	0.973	1.000	0.991	1.009
EGFR	0.000	0.003	0.001	0.975	1.000	0.993	1.007
STAT3P	0.000	0.005	0.000	0.984	1.000	0.991	1.009

Abbreviations: HR, hazard ratio (i.e. exp(β)) for a one-unit change of a variable; $^{0.95}CI_{LB}$ - $^{0.95}CI_{UB}$,lower and upper bound of the 95% confidence interval of HR.; β, regression coefficient a variable in the Cox proportional hazard regression model; SE, standard error of β coefficient; Wald, value of the Wald test; *p* ,probability of the Wald test.
